# Supplementary material for: Identifying Predictor Variables for a Composite Risk Prediction Tool for Gestational Diabetes and Hypertensive Disorders of Pregnancy: A Modified Delphi Study
Source: Healthcare (Basel). 2024 Jul 8;12(13):1361. doi: 10.3390/healthcare12131361 (PMC11241067; doi:10.3390/healthcare12131361)
Supplement: Supplementary file 1 [file healthcare-12-01361-s001.zip › healthcare-3058597-supplementary.pdf]

Identifying predictor variables for a composite risk prediction tool for Gestational Diabetes and Hypertensive Disorders of Pregnancy: a modified Delphi study

Stephanie Cowan[1]*, Sarah Lang[1]*, Rebecca Goldstein[1,2], Joanne Enticott[1], Frances Taylor[1], Helena Teede[1,2], Lisa Moran[1,3]
*Joint First Author

Author Affiliations

1. Monash Centre for Health Research and Implementation, School of Public Health and Preventive Medicine, Monash University, Clayton, VIC 3168, Australia
2. Monash Endocrine and Diabetes Units, Monash Health, Clayton, Melbourne, Victoria, Australia
3. Victorian Heart Institute, Monash Health, Clayton, Melbourne, Victoria, Australia

Corresponding Author
Associate Professor Lisa Moran
Address: Monash Centre for Health Research and Implementation, School of Public Health and Preventive Medicine, Monash University, Clayton, VIC 3168, Australia
Telephone: +61 3 8572 2600
Email: lisa.moran@monash.edu

Supporting Information

Table of Contents

Table S1 - Risk prediction models for Gestational Diabetes Mellitus used to inform the list of predictors for consideration by the expert panel ... 2

Table S2 - Risk prediction models for De Novo Gestational Hypertension and Preeclampsia used to inform the list of predictors for consideration by the expert panel ... 5

Table S3 – Clinical practice guidelines reviewed for Gestational Diabetes and Hypertensive Disorders of Pregnancy to inform the list of predictors for consideration by the expert panel ... 9

Table S4 - Basic definitions of predictor variables ranked in the *Rounds One and Two* of the modified e-Delphi process ... 10

Table S5 - Additional predictor variables presented to panellists for consideration[a] ... 12

Table S6 - Overview of the online consensus meeting ... 13

Figure S1 – Flowchart of Delphi process and panellist recruitment ... 14

References ... 15

Table S1 - Risk prediction models for Gestational Diabetes Mellitus used to inform the list of predictors for consideration by the expert panel

Author (Year)	Country	Sample size	Data collection time points	Externally validated (Y/N)	Predictor variables
**Identified by Thong et al.[1] narrative review[a]**					
**Adam (2017)[2]**	South Africa	554	Screening at recruitment (<26 weeks) and OGTT second trimester (24-28 weeks)	N	FBG, BMI, Hx foetal macrosomia, HbA1c
**Benhalima (2020)[3]**	Belgium	1843	Screening first trimester (6-14 weeks) and OGTT second trimester (24-28 weeks)	N	Family Hx diabetes, Hx smoking, Hx GDM, Ethnicity/race, Age, Height, FBG, TG, HbA1C
**Capula (2016)[4]**	Italy	3974	Screening second trimester (16-18 weeks) and OGTT second trimester (24-28 weeks)	N	Hx GDM
**Donovan (2019)[5]**	USA	1156708	Retrospectively collected from medical records (no specific timepoint(s) given)	Y	Race/ethnicity, BMI, Age, Hx chronic hypertension
**Gao (2020)[6]**	China	19331	Screening at registration (≤15 weeks) and OGTT second trimester (24-28 weeks)	N	Age, BMI, Height, SBP, ALT, Family Hx diabetes, Physical activity during pregnancy, Sitting time during pregnancy, GWG, Hx smoking
**Guo (2020)[7]**	China	3956	Screening first trimester (9-13 weeks) and OGTT second trimester (24-28 weeks)	N	Age, BMI, FGB, Family Hx diabetes
**Schaefer (2018)[8]**	China	1129	Screening second trimester (14-20 weeks) and OGTT second trimester (24-28 weeks)	N	Age, Family Hx diabetes, BMI, GWG, Hx GDM
**Schoenaker (2018)[9]**	Australia	6504	Screening preconception and GDM diagnosis was self-reported (using retrospective questionnaire data)	N	Age at menarche, Ethnicity/race, Family Hx diabetes, Proposed age at future first pregnancy, BMI, Diet, Physical activity, Hx PCOS
**Snyder (2020)[10]**	USA	105379	Screening first trimester (10-14 weeks), second trimester (15-	N	Ethnicity/race, Age, BMI, Family Hx diabetes, Hx chronic hypertension, PAPP-A, Unconjugated estriol, Dimeric inhibin A

Author (Year)	Country	Sample size	Data collection time points	Externally validated (Y/N)	Predictor variables
			20 weeks) and OGTT timing not reported		
**Sweeting (2017)[11]**	Australia	980	Screening first trimester (11+0-13+6 weeks) and OGTT second trimester (24-28 weeks)	N	Hx GDM, Family Hx diabetes, BMI, Age, Ethnicity/race, Parity
**Sweeting (2018)[12]**	Australia	986	Screening first trimester (11+0-13+6 weeks) and OGTT second trimester (24-28 weeks)	N	Hx GDM, Parity, Family Hx diabetes, Ethnicity/race, BMI, MAP, UTA PI, PAPP-A
**Sweeting (2019)[13]**	Australia	986	Screening first trimester (11+0-13+6 weeks) and OGTT second trimester (24-28 weeks)	N	Hx GDM, Parity, Family Hx diabetes, Ethnicity/race, BMI, MAP, UTA PI, PAPP-A, Free β-human chorionic gonadotropin, Lipocalin-2 (adipogenic marker), TG
**Theriault (2016)[14]**	Canada	792	Screening second trimester (14-17 weeks) and OGTT second trimester (24-28 weeks)	N	HbA1c, SHBG, BMI, Family Hx diabetes, Hx GDM, Diet
**Van Hoorn (2021)[15]**	Netherlands	1073	Screening first trimester (week of gestation not reported) and second trimester (24-28 weeks)	N	Age, BMI, Ethnicity/race, Family Hx diabetes, Hx GDM, FBG
**White (2016)[16]**	United Kingdom	1303	Screening second trimester (15+0-18+6 weeks) and OGTT second and third trimester (23+0-32+6 weeks)	N	Age, Hx GDM, Family Hx T2DM, SBP, Sum of skinfold thicknesses, Waist: height and neck: thigh ratios, PCOS, Age, FBG, BMI
**Ye (2020)[17]**	China	22242	Screening second trimester (16-20 weeks) and OGTT second trimester (24-28 weeks)	N	FBG, Hba1c, TG, BMI, HbA1c, HDL-C, Hx GDM, Hx preterm birth
**Zhang (2020)[18]**	China	1385	Screening first trimester (7-12 weeks) and OGTT second trimester (24-28 weeks)	N	Age, BMI, Hx chronic hypertension, HbA1C, TC, TG, Parity
**Zheng (2019)[19]**	China	4771	Screening first and second trimester (8-20 weeks) and OGTT second trimester (22-26 weeks)	N	Age, BMI, FBG, TG, TC, LDL-C
**Additional contemporary models identified**					

Author (Year)	Country	Sample size	Data collection time points	Externally validated (Y/N)	Predictor variables
**Nombo (2018)**[20]	Tanzania	609	Screening and OGTT second and third trimester (20-38 weeks)	N	Mid-upper arm circumference ≥28 cm, Hx stillbirth, Family Hx T2DM
**Cooray**[b] **(2021)**[21]	Australia	26474	Screening first trimester (12-15 weeks) and OGTT second trimester (26-28 weeks)	Y	Age, BMI, Ethnicity/race, Family Hx diabetes, Hx GDM, Hx macrosomic baby, Hx shoulder dystocia, Hx PE

[a] Searched from 2016 to June 2021, as an update to the review by Kenelly et al. on risk prediction models in GDM, published in 2016.

[b] This is an update of an earlier risk prediction model by Teede et al.[22]

ABBREVIATIONS: ALT: Alanine transaminase, APS: Antiphospholipid syndrome, AST: Aspartate transaminase, Beta-hCG: Beta human choriogonadotropin, BMI: Body mass index, BUN: Blood urea ratio, CI: Cardiac output index, CYC: Cystatin C, DBP: Diastolic blood pressure, FBG: Fasting blood glucose, GDM: Gestational diabetes mellitus, GH: Gestational hypertension, GWG: Gestational weight gain, HDL-C: high density lipoprotein cholesterol, Hx: History, LDL-C: low density lipoprotein cholesterol, MAP: Mean arterial pressure, NGAL: Neutrophil gelatinase-associated lipocalin, PAPP-A: Pregnancy-associated plasma protein-A, PE: Preeclampsia, PIGF: Placental growth factor, PCOS: Polycystic Ovary Syndrome, SHBG: sex hormone binding globulin, SBP: Systolic blood pressure, SBPAO: Systolic blood pressure in the aorta,sFlt-1: Soluble fms-like tyrosine kinase-1SLE: Systemic lupus erythematosus, T1DM: Type 1 diabetes mellitus, T2DM: Type 2 diabetes mellitus, TBA: Total bile acid, TCO_2: Total carbon dioxide in blood, TG: Triglycerides, TPR: Total peripheral resistance, UACR: Urine albumin-creatinine ratio, UPCR: Urine protein-creatinine ratio, UTA PI: Uterine artery pulsatility index, WBC: White blood cell count

Table S2 - Risk prediction models for De Novo Gestational Hypertension and Preeclampsia used to inform the list of predictors for consideration by the expert panel

Author (Year)	Country	Sample size	Outcome(s)	Predictor variable collection time point(s)	Externally validated (Y/N)	Predictor variables
**Identified by Thong et al.[1] narrative review[a]**						
**Sepúlveda-Martínez (2019)[23]**	Chile	1756	PE (pre-term delivered <37 weeks and term delivered ≥37 weeks)	First trimester (11+0-13+6 weeks)	N	MAP, UTA PI, Hx smoking, BMI, Age, Hx PE, Chronic HT, Hx SLE/APS, Parity
**Lewandowska (2020)[24]**	Poland	912	PE and isolated GH	First trimester (10-14 weeks) and throughout pregnancy	N	BMI, Age, Lower financial status, Level of education, Hx isolated GH, Primiparity, GWG, Lack of multivitamin supplements 2nd-3rd trimester, Interpregnancy interval
**Mula (2019)[25]**	Spain	1777	PE (late delivered ≥34 weeks)	First trimester (11+0-13+6 weeks), second trimester (20+0-22+6 weeks), third trimester (32+0-33+6 weeks)	N	MAP, UTA PI, Hx kidney disease
**Yang (2020)[26]**	China	690	HDP (not defined)	First trimester (≤13 weeks), second trimester (14-20 and 21–27 weeks), third trimester (28–34 and ≥35 weeks)	Y	MAP, SBP, DBP, Total peripheral resistance (TPR), Cardiac output index (CI), Pulse pressure, Platelets, Haematocrit, Creatinine, Uric acid, BMI, Hx spontaneous abortion/miscarriage, Multiple pregnancy
**Serra (2020)[27]**	Spain	6893	PE (early delivered <34 weeks)	First trimester (8+0/7-13+6/7 weeks)	N	MAP, UTA PI, PIGF, Age, Ethnicity/race, Hx PE, Hx chronic hypertension, Parity
**Hyun Jhee (2019)[28]**	Korea	11006	PE (late occurring ≥34 weeks)	Second trimester (14-17 weeks) through to third trimester (34 weeks)	N	SBP, Platelets, WBC, Creatinine, BUN, UACR, UPCR, Potassium, Calcium, Magnesium, AST, ALT, TCO2, Body weight, Gestational age

Author (Year)	Country	Sample size	Outcome(s)	Predictor variable collection time point(s)	Externally validated (Y/N)	Predictor variables
**Sovio (2019)[29]**	United Kingdom	4184	PE (pre-term delivered <37 weeks)	First trimester (12 weeks), second trimester (20 weeks), third trimester (28 and 36 weeks)	N	Age, Height, Body weight, Ethnicity/race, Hx chronic hypertension, Hx SLE/APS, Family Hx PE, Hx T1DM, Hx T2DM, Conception method
**Hou (2020)[30]**	China	316	De novo GH	Second trimester (16-20 weeks)	N	Uric acid, Homocysteine, TBA, CYC, BMI, Age
**Allen (2017)[31]**	United Kingdom	1045	PE and de novo GH	First trimester (11–14 weeks)	N	MAP, SBP in the aorta, Body weight
**Schaller[b] (2020)[32]**	Germany	5413	PE (late occurring ≥34 weeks)	First trimester (11+0-13+6 weeks)	Y	MAP, UTA PI, BMI, Age, Ethnicity/race, Hx PE, Family Hx PE
**Murtoniemi (2018)[33]**	Finland	164	PE (also looks at subsets early, late, severe and non-severe)	First trimester (11-13 weeks)	N	MAP, UTA PI, PAPP-A, PIGF, Beta-hCG, BMI, Age, Primiparity
**Al-Rubaie (2020)[34]**	Australia	12395	PE	From first antenatal visit to discharge (no specific timepoint given)	Y	BMI, Age, Ethnicity/race, Hx chronic hypertension, Hx SLE/APS, Family Hx PE, Multiple pregnancy
**Antwi (2018)[35]**	Ghana	373	De novo GH	First trimester (8-13 weeks)	N	DBP, PAPP-A, PIGF, Height, Body weight, Hx GH, Family Hx hypertension, Parity
**Pihl (2019)[36]**	Denmark	14207	PE (pre-term delivered <37 weeks and term delivered ≥37 weeks)	First trimester (median 11+0 (range 8+1-13+5) weeks)	N	MAP, PIGF, NGAL, BMI
**Sandstrom (2019)[37]**	Sweden	62562	PE (delivery <34, <37, and ≥37 weeks)	First antenatal visit (no specific timepoint given)	N	MAP, Haemoglobin, Protein in urine, FBG, Hx smoking, Alcohol use, BMI, Age, Ethnicity/race, Family situation, Blood group, Hx kidney disease, Hx diabetes, Hx hepatitis, Hx crohns/ulcerative colitis, Hx psychiatric disease,

Author (Year)	Country	Sample size	Outcome(s)	Predictor variable collection time point(s)	Externally validated (Y/N)	Predictor variables
						Family Hx hypertension, Family history PE, Gestational age at first antenatal visit, Prior infertility, Conception method
**Additional contemporary models identified**						
**Allotey[c] (2020)[38]**	International cohort (United Kingdom, United States, Australia, New Zealand, Ireland, Argentina, Colombia, Peru, India, Italy, Kenya, Switzerland and Thailand)	20132	PE (early <34 weeks, late ≥34 weeks and any-onset)	All three trimesters (different time points used across cohorts)	N	SBP, PIGF, sFlt-1, Age, BMI, Hx PE, Hx chronic hypertension, Hx diabetes, Hx kidney disease, Parity
**Earlier models identified through current national and international guidelines[d]**						
**O'Gorman (2016)[39]**	United Kingdom	35948	PE (pre-term delivered <37 weeks and term delivered ≥37 weeks)	First trimester (11-13 weeks)	Y	MAP, UTA PI, PAPP-A, PIGF, Hx smoking, Height, Body weight, Age, Ethnicity/race, Hx chronic hypertension, Hx SLE/APS, Hx T1DM, Hx T2DM, Family Hx PE, Singleton or multiple pregnancy, Parity, Conception method, Gestational age, Medication(s) if

Author (Year)	Country	Sample size	Outcome(s)	Predictor variable collection time point(s)	Externally validated (Y/N)	Predictor variables
						Hx of T2DM, Interpregnancy interval, Gestation at delivery (from previous births for women who are parous)

[a] The literature search included articles from 2018 to June 2021, to reflect the updates in HDP classification and diagnosis

[b] This model combines three earlier algorithms and is therefore an external validation of these earlier models

[c] Uses individual participant data from four cohort studies

[d] Promoted for early screening of PE by the International Federation of Gynaecology and Obstetrics (FIGO) initiative on preeclampsia: A pragmatic guide for first-trimester screening and prevention

ABBREVIATIONS: ALT: Alanine transaminase, APS: Antiphospholipid syndrome, AST: Aspartate transaminase, Beta-hCG: Beta human choriogonadotropin, BMI: Body mass index, BUN: Blood urea ratio, CI: Cardiac output index, CYC: Cystatin C, DBP: Diastolic blood pressure, FBG: Fasting blood glucose, GH: Gestational hypertension, GWG: Gestational weight gain, Hx: History, MAP: Mean arterial pressure, NGAL: Neutrophil gelatinase-associated lipocalin, PAPP-A: Pregnancy-associated plasma protein-A, PE: Preeclampsia, PIGF: Placental growth factor, SBP: Systolic blood pressure, sFlt-1: Soluble fms-like tyrosine kinase-1SLE: Systemic lupus erythematosus, T1DM: Type 1 diabetes mellitus, T2DM: Type 2 diabetes mellitus, TBA: Total bile acid, TCO_2: Total carbon dioxide in blood, TPR: Total peripheral resistance, UACR: Urine albumin-creatinine ratio, UPCR: Urine protein-creatinine ratio, UTA PI: Uterine artery pulsatility index, WBC: White blood cell count

Table S3 – Clinical practice guidelines reviewed for Gestational Diabetes and Hypertensive Disorders of Pregnancy to inform the list of predictors for consideration by the expert panel

Country	GDM Guidelines	HDP Guidelines
**Australia and New Zealand**	Queensland Clinical Guidelines - Gestational Diabetes Mellitus (GDM)	Queensland Clinical Guidelines - Hypertension and Pregnancy
	Australasian Diabetes in Pregnancy Society (ADIPS) - Consensus Guidelines for the Testing and Diagnosis of Hyperglycaemia in Pregnancy in Australia and New Zealand	Society of Obstetric Medicine Australian and New Zealand (SOMANZ) - Guidelines for the management of hypertensive disorders of pregnancy 2014
	Australian Government Department of Health, Pregnancy Care Guidelines – Clinical Practice Guidelines Pregnancy Care	Australian Government Department of Health, Pregnancy Care Guidelines – Clinical Practice Guidelines Pregnancy Care
**Canada**	Society of Obstetricians and Gynaecologists of Canada - Guideline No. 393-Diabetes in Pregnancy	Society of Obstetricians and Gynaecologists of Canada - Diagnosis, Evaluation, and Management of the Hypertensive Disorders of Pregnancy: Executive Summary
**European**		European Society of Cardiology - 2018 ESC Guidelines for the management of cardiovascular diseases during pregnancy
**United Kingdom**	National Institute for Health and Care Excellence - Guidelines, Diabetes in pregnancy: management from preconception to the postnatal period	National Institute for Health and Care Excellence - Hypertension in Pregnancy: Diagnosis and Management
**United States**	American College of Obstetricians and Gynecologists - Practice Bulletin No. 190: Gestational Diabetes Mellitus	American College of Obstetricians and Gynecologists - Gestational Hypertension and Preeclampsia: ACOG Practice Bulletin, Number 222
	American Diabetes Association Professional Practice C. 2. Classification and Diagnosis of Diabetes: Standards of Medical Care in Diabetes -2022	Screening for Preeclampsia: US Preventive Services Task Force Recommendation Statement
**International**	Endocrine Society - Endocrine Society, Diabetes and Pregnancy: An Endocrine Society Clinical Practice Guideline	The International Society for the Study of Hypertension - Hypertensive Disorders of Pregnancy: ISSHP Classification, Diagnosis, and Management Recommendations for International Practice
	The International Federation of Gynecology and Obstetrics (FIGO) Initiative on gestational diabetes mellitus: A pragmatic guide for diagnosis, management, and care	The International Federation of Gynaecology and Obstetrics (FIGO) initiative on pre-eclampsia: A pragmatic guide for first-trimester screening and prevention
	International Association of Diabetes and Pregnancy Study Groups (IADPSG) - Recommendations on the Diagnosis and Classification of Hyperglycaemia in Pregnancy	International Society of Ultrasound in Obstetrics and Gynecology - ISUOG Practice Guidelines: role of ultrasound in screening for and follow-up of pre-eclampsia
	World Health Organisation, Diagnostic Criteria and Classification of Hyperglycaemia First Detected in Pregnancy	

Table S4 - Basic definitions of predictor variables ranked in the *Rounds One and Two* of the modified e-Delphi process

Predictor variable	Basic definition
**Maternal Demographics**	
Age[a]	Maternal age in years
Ethnicity[a]	Race is usually associated with biology and linked with certain phenotypic presentations. Ethnicity is more broadly defined as categorising people according to common national, tribal, religious, linguistic, cultural origin or background (e.g. linked with cultural expression and identification)
Postcode (proxy SES)[b]	Area of residence denoted by a group of numbers and/or letters such as a postcode or ZIP code
**Pre-pregnancy History**	
Height[a]	Maternal height in cm or feet and inches
Body mass index[a]	Internationally recognised standard used to determine whether people are in a healthy weight range for their height, defined as the body mass divided by square of body height (expressed in units of kg/m^2)
History smoking[a]	Self-reported, either during or before pregnancy
Pre-existing diabetes (type one and two diabetes mellitus)[b]	Type one or type two diabetes mellitus onset or first recognition occurring prior to conception
History polycystic ovary syndrome[b]	Diagnosed through the European Society for Human Reproduction and Embryology/American Society for Reproductive Medicine (ESRHE/ASRM) criteria, requiring two of the following features: polycystic ovaries on ultrasound, oligoovulatory or anovulatory cycles and biochemical or clinical hyperandrogenism
History chronic kidney disease[b]	The presence of impaired or reduced kidney function lasting at least 3 months
Waist circumference[b]	The circumference of the abdomen at its narrowest point between the lower costal (10th rib) border and the top of the iliac crest perpendicular to the long access of the trunk
Family history diabetes (including type one or two diabetes mellitus, and gestational diabetes mellitus)[b]	First degree relative with type one or two diabetes, and mother or sister who experienced gestational diabetes mellitus during pregnancy
Family history hypertension[b]	First degree relative with chronic hypertension, and mother or sister who experienced hypertensive disorders of pregnancy, including gestational hypertension and preeclampsia-eclampsia
**Obstetric History**	
Gestational weight gain[a]	Weight gained during pregnancy, commonly defined as the difference between the final pregnancy weight and pre-pregnancy weight. The pre-pregnancy weight may be self-reported, or measured at the first antenatal visit

Predictor variable	Basic definition
Pre-existing (chronic) hypertension[a]	High blood pressure that was present before pregnancy or that occurs before 20 weeks of pregnancy
Parity[a]	Total number of previous pregnancies experienced that have resulted in a live birth or a stillbirth. Risk predictor data will be inputted as nulliparous (hasn't given birth) or parous (has given birth)
History preeclampsia[a]	Diagnosed by the presence of de novo hypertension after 20 weeks' gestation, accompanied by proteinuria and/or evidence of maternal acute kidney injury, liver dysfunction, neurological features, haemolysis or thrombocytopenia, or foetal growth restriction
History gestational diabetes mellitus[b]	A condition characterized by an elevated level of glucose in the blood during pregnancy, with onset or first recognition occurring during pregnancy, typically resolving after the birth
History macrosomia[b]	Baby with birth weight > 4500g or > 90th centile
**Biochemical Measures**	
Blood glucose levels[a]	The concentration of glucose in the blood
Pregnancy-associated plasma protein-A[a]	A complex, high molecular weight glycoprotein that can be used as a screening marker for Down syndrome. Having a low level in the first trimester is considered a risk factor for having a small for gestation age baby
Alanine transaminase[a]	An enzyme found primarily in the liver, measured in the serum that is used as a biochemical marker of liver injury
**Haemodynamic Measures**	
Mean arterial pressure[a]	Average of blood pressure over cardiac cycle that can be used as a predictor for adverse cardiovascular outcomes. This can be estimated by a formula (diastolic pressure plus one third of difference between diastolic and systolic pressure)
Uterine artery pulsatility index[a]	This is a measurement of the flow in the uterine arteries in a doppler ultrasound which has the potential to predict pregnancy complications associated with uteroplacental insufficiency. Resistance to blood flow within the uteroplacental circulation is transmitted upstream to the uterine arteries and can be measured as increased pulsatility index
Systolic blood pressure[a]	Blood pressure during contraction of the ventricles, measured in mm Hg

[a] Predictor variable included in the *Round One* Delphi survey

[b] Predictor variable nominated for further consideration in the *Round Two* consensus meeting

Table S5 - Additional predictor variables presented to panellists for consideration[a]

**Predictor variable**[b]
**Maternal Demographics**
No additional predictor variables nominated
**Pre-pregnancy History**
Family history diabetes (including type one or two diabetes mellitus, and gestational diabetes mellitus
Family history hypertension (including chronic hypertension and hypertensive disorders of pregnancy)
History of systemic lupus erythematosus/antiphospholipid syndrome
History polycystic ovary syndrome
Medications used to treat type two diabetes mellitus
**Obstetric History**
Conception method (i.e. spontaneous, ovulation drugs, in vitro fertilisation)
Singleton or multiple pregnancy
Gestational age
History pregnancy complications (includes macrosomic baby and shoulder dystocia)
History spontaneous abortion/miscarriage
Interpregnancy interval
History of gestation at delivery (from previous births for women who are parous)
**Biochemical Measures**
Placental growth factor
HbA1c
Triglycerides
Platelets
Haematocrit
Creatinine
Uric acid
**Haemodynamic Measures**
Diastolic blood pressure
Total peripheral resistance
Cardiac output index
Pulse pressure

[a] Survey respondents were asked to nominate any additional predictor variables not included in the *Round One* survey that they deemed important for further consideration in the *Round Two* consensus meeting.

[b] These 24 predictor variables were provided as good candidates for further consideration on the basis that they were used frequently in existing risk prediction models (at least 15% of models) or were used in models that had undergone external validation.

Table S6 - Overview of the online consensus meeting

**CONSENSUS MEETING ACTIVITY**	**ACTIVITY AIM**
Participant Arrival	-
Presentation of the workshop aims, review findings, *Round One* survey results and instructions for the small group discussions.	• To introduce the workshop aims.<br>• To communicate the results of *Round One.*
Small group discussion facilitated using a nominal group technique. Three to four predictor variables were allocated to each group for in-depth discussion.	• To discuss the definition of each variable.<br>• To discuss the *clinical importance* of each predictor variable in predicting the risk of GDM or HDP.<br>• To discuss the *feasibility* of collecting each predictor variable in early pregnancy across varied clinical settings.<br>• To discuss the *acceptability* of collecting each predictor variable in early pregnancy.
Feedback from small group discussion to the broader panel, followed by large group discussion.	
Participants independently and anonymously complete *the Round Two* online survey.	• To prioritise predictor variables that are *clinically important* in identifying women at risk of HDP or GDM, *feasible* and *acceptable* to collect in early pregnancy for inclusion in a composite risk prediction tool.
BREAK (Results synthesis)	• To synthesise the results from the *Round Two* survey
Presentation of *Round Two* results.	-
Large group discussion of variables prioritised for inclusion in the risk prediction tool.	• To obtain consensus regarding the predictor variables to include in the composite risk prediction tool for cardiometabolic risk during pregnancy.
Questions and final comments	-
**POST-CONSENSUS MEETING**	**ACTIVITY AIM**
Final list of predictor variables emailed to panellists.	• To facilitate independent reflection and comment on the finalised predictor variables for inclusion in the composite risk prediction tool.

Abbreviations: Gestational Diabetes (GDM), Hypertensive Disorders of Pregnancy (HDP)

Figure S1 – Flowchart of Delphi process and panellist recruitment

Recruitment of Delphi Panellists	
**Experts and Clinicians (n=61)**<br>▪ Recruitment via Purposive Sampling using email	**Consumers with lived experience (n=6)**<br>▪ Recruitment from existing Consumer Advisory Committees that advocate for women in healthcare research using email

↓

Round One: E-Delphi Round One Online Survey (N=31)	
**Experts and Clinicians (n=31)**<br>▪ Obstetrics and gynaecology (n=7)<br>▪ Endocrinologist (n=6)<br>▪ Midwife (n=5)<br>▪ Dietitian (n=5)<br>▪ Epidemiologist (n=3)<br>▪ Biostatistician (n=2)<br>▪ Health economist (n=1)<br>▪ General practitioner (n=1)<br>▪ Did not disclose (n=1)	**Consumers with lived experience (n=0)**<br>*Consumers were not invited to participate in the *Round 1* Survey

↓

Invited to Attend Virtual Consensus Meeting (n=37)	
**Experts and Clinicians (n=31)**<br>▪ Experts and Clinicians who participated in *Round 1*	**Consumers with lived experience (n=6)**<br>▪ Women with prior GDM and HDP

→

Unable to Attend Virtual Consensus Meeting/ Did Not Continue (n=23)
**Experts and Clinicians (n=19)**<br>▪ Obstetrics and gynaecology (n=6)<br>▪ Endocrinologist (n=2)<br>▪ Midwife (n=4)<br>▪ Dietitian (n=3)<br>▪ Epidemiologist (n=1)<br>▪ Biostatistician (n=1)<br>▪ Health economist (n=1)<br>▪ Did not disclose (n=1)<br><br>**Consumers with lived experience (n=4)**

↓

Consensus Meeting and Round Two Online Survey (n=14)	
**Experts and Clinicians (n=12)**<br>▪ Obstetrics and gynaecology (n=1)<br>▪ Endocrinologist (n=4)<br>▪ Midwife (n=1)<br>▪ Dietitian (n=2)<br>▪ Epidemiologist (n=2)<br>▪ Biostatistician (n=1)<br>▪ General practitioner (n=1)	**Consumers with lived experience (n=2)**<br>▪ Consumers with prior GDM (n=2)

References

1. Thong, E.P.; Ghelani, D.P.; Manoleehakul, P.; Yesmin, A.; Slater, K.; Taylor, R.; Collins, C.; Hutchesson, M.; Lim, S.S.; Teede, H.J.; et al. Optimising Cardiometabolic Risk Factors in Pregnancy: A Review of Risk Prediction Models Targeting Gestational Diabetes and Hypertensive Disorders. *J. Cardiovasc. Dev. Dis.* **2022**, *9*, 55. https://doi.org/10.3390/jcdd9020055.
2. Adam, S.; Rheeder, P. Selective screening strategies for gestational diabetes: A prospective cohort observational study. *J. Diabetes Res*. **2017**, *2017*, 2849346. https://doi.org/10.1155/2017/2849346.
3. Benhalima, K.; Van Crombrugge, P.; Moyson, C.; Verhaeghe, J.; Vandeginste, S.; Verlaenen, H.; Vercammen, C.; Maes, T.; Dufraimont, E.; De Block, C.; et al. Estimating the risk of gestational diabetes mellitus based on the 2013 WHO criteria: A prediction model based on clinical and biochemical variables in early pregnancy. *Acta Diabetol*. **2020**, *57*, 661–671. https://doi.org/10.1007/s00592-019-01469-5.
4. Capula, C.; Chiefari, E.; Borelli, M.; Oliverio, R.; Vero, A.; Foti, D.; Puccio, L.; Vero, R.; Brunetti, A. A new predictive tool for the early risk assessment of gestational diabetes mellitus. *Prim. Care Diabetes* **2016**, *10*, 315–323. https://doi.org/10.1016/j.pcd.2016.05.004.
5. Donovan, B.M.; Breheny, P.J.; Robinson, J.G.; Baer, R.J.; Saftlas, A.F.; Bao, W.; Greiner, A.L.; Carter, K.D.; Oltman, S.P.; Rand, L.; et al. Development and validation of a clinical model for preconception and early pregnancy risk prediction of gestational diabetes mellitus in nulliparous women. *PLoS ONE*. **2019**, *14*, e0215173. https://doi.org/10.1371/journal.pone.0215173.
6. Gao, S.; Leng, J.; Liu, H.; Wang, S.; Li, W.; Wang, Y.; Hu, G.; Chan, J.C.N.; Yu, Z.; Zhu, H.; et al. Development and validation of an early pregnancy risk score for the prediction of gestational diabetes mellitus in Chinese pregnant women. *BMJ Open Diabetes Res. Care*. **2020**, *8*, e000909. https://doi.org/10.1136/bmjdrc-2019-000909.
7. Guo, F.; Yang, S.; Zhang, Y.; Yang, X.; Zhang, C.; Fan, J. Nomogram for prediction of gestational diabetes mellitus in urban, Chinese, pregnant women. *BMC Pregnancy Childbirth*. **2020**, *20*, 1–8. https://doi.org/10.1186/s12884-019-2703-y.
8. Schaefer, K.K.; Xiao, W.; Chen, Q.; He, J.; Lu, J.; Chan, F.; Chen, N.; Yuan, M.; Xia, H.; Lam, K.B.H.; et al. Prediction of gestational diabetes mellitus in the Born in Guangzhou Cohort Study, China. *Int. J. Gynaecol. Obstet*. **2018**, *143*, 164–171. https://doi.org/10.1002/ijgo.12627.
9. Schoenaker, D.A.; Vergouwe, Y.; Soedamah-Muthu, S.S.; Callaway, L.K.; Mishra, G.D. Preconception risk of gestational diabetes: Development of a prediction model in nulliparous Australian women. *Diabetes Res. Clin. Pract*. **2018**, *146*, 48–57. https://doi.org/10.1016/j.diabres.2018.09.021.
10. Snyder, B.M.; Baer, R.J.; Oltman, S.P.; Robinson, J.G.; Breheny, P.J.; Saftlas, A.F.; Bao, W.; Greiner, A.L.; Carter, K.D.; Rand, L.; et al. Early pregnancy prediction of gestational diabetes mellitus risk using prenatal screening biomarkers in nulliparous women. *Diabetes Res. Clin. Pract*. **2020**, *163*, 108139. https://doi.org/10.1016/j.diabres.2020.108139.
11. Sweeting, A.N.; Appelblom, H.; Ross, G.P.; Wong, J.; Kouru, H.; Williams, P.F.; Sairanen, M.; Hyett, J.A. First trimester prediction of gestational diabetes mellitus: A clinical model based on maternal demographic parameters. *Diabetes Res. Clin. Pract*. **2017**, *127*, 44-50. https://doi.org/10.1016/j.diabres.2017.02.036.
12. Sweeting, A.N.; Wong, J.; Appelblom, H.; Ross, G.P.; Kouru, H.; Williams, P.F.; Sairanen, M.; Hyett, J.A. A first trimester prediction model for gestational diabetes utilizing aneuploidy and pre-eclampsia screening markers. *J. Matern.-Fetal Neonatal Med.* **2018**, *31*, 2122–2130.
13. Sweeting, A.N.; Wong, J.; Appelblom, H.; Ross, G.P.; Kouru, H.; Williams, P.F.; Sairanen, M.; Hyett, J.A. A novel early pregnancy risk prediction model for gestational diabetes mellitus. *Fetal Diagn. Ther*. **2019**, *45*, 76–84. https://doi.org/10.1159/000486853.
14. Thériault, S.; Giguère, Y.; Massé, J.; Girouard, J.; Forest, J.-C. Early prediction of gestational diabetes: A practical model combining clinical and biochemical markers. *Clin. Chem. Lab. Med*. **2016**, *54*, 509–518. https://doi.org/10.1515/cclm-2015-0537.
15. van Hoorn, F.; Koster, M.P.; Kwee, A.; Groenendaal, F.; Franx, A.; Bekker, M.N. Implementation of a first-trimester prognostic model to improve screening for gestational diabetes mellitus. *BMC Pregnancy Childbirth*. **2021**, *21*, 1–11. https://doi.org/10.1186/s12884-021-03749-x.
16. White, S.L.; Lawlor, D.A.; Briley, A.L.; Godfrey, K.M.; Nelson, S.M.; Oteng-Ntim, E.; Robson, S.C.; Sattar, N.; Seed, P.T.; Vieira, M.C.; et al. Early antenatal prediction of gestational diabetes in obese women: Development of prediction tools for targeted intervention. *PLoS ONE* **2016**, *11*, e0167846. https://doi.org/10.1371/journal.pone.0167846.

17. Ye, Y.; Xiong, Y.; Zhou, Q.; Wu, J.; Li, X.; Xiao, X. Comparison of machine learning methods and conventional logistic regressions for predicting gestational diabetes using routine clinical data: A retrospective cohort study. *J. Diabetes Res*. **2020**, *2020*, 4168340. https://doi.org/10.1155/2020/4168340.
18. Zhang, X.; Zhao, X.; Huo, L.; Yuan, N.; Sun, J.; Du, J.; Nan, M.; Ji, L. Risk prediction model of gestational diabetes mellitus based on nomogram in a Chinese population cohort study. *Sci. Rep*. **2020**, *10*, 1–7. https://doi.org/10.1038/s41598-020-78164-x.
19. Zheng, T.; Ye, W.; Wang, X.; Li, X.; Zhang, J.; Little, J.; Zhou, L.; Zhang, L. A simple model to predict risk of gestational diabetes mellitus from 8 to 20 weeks of gestation in Chinese women. *BMC Pregnancy Childbirth*. **2019**, *19*, 1–10. https://doi.org/10.1186/s12884-019-2374-8.
20. Nombo, A.P.; Mwanri, A.W.; Brouwer-Brolsma, E.M.; Ramaiya, K.L.; Feskens, E.J. Gestational diabetes mellitus risk score: A practical tool to predict gestational diabetes mellitus risk in Tanzania. *Diabetes Res. Clin. Pract*. **2018**, *145*, 130-137. https://doi.org/10.1016/j.diabres.2018.05.001.
21. Cooray, S.D.; De Silva, K.; Enticott, J.; Dawadi, S.; Boyle, J.A.; Soldatos, G.; Paul, E.; Versace, V.; Teede, H.J. External validation and updating of a prediction model for the diagnosis of gestational diabetes mellitus. *medRxiv* **2021**. https://doi.org/10.1101/2021.12.05.21267329.
22. Teede, H.J.; Harrison, C.L.; Teh, W.T.; Paul, E.; Allan, C.A. Gestational diabetes: Development of an early risk prediction tool to facilitate opportunities for prevention. *Aust. N. Z. J. Obstet. Gynaecol*. **2011**, *51*, 499–504. https://doi.org/10.1111/j.1479-828X.2011.01356.x.
23. Sepúlveda-Martínez, A.; Rencoret, G.; Silva, M.C.; Ahumada, P.; Pedraza, D.; Muñoz, H.; Valdés, E.; Parra-Cordero, M. First trimester screening for preterm and term pre-eclampsia by maternal characteristics and biophysical markers in a low-risk population. *J. Obstet. Gynaecol. Res*. **2019**, *45*, 104–112. https://doi.org/10.1111/jog.13809.
24. Lewandowska, M.; Więckowska, B.; Sajdak, S.; Lubiński, J. Pre-pregnancy obesity vs. other risk factors in probability models of preeclampsia and gestational hypertension. *Nutrients* **2020**, *12*, 2681. https://doi.org/10.3390/nu12092681.
25. Mula, R.; Meler, E.; Albaiges, G.; Rodriguez, I. Strategies for the prediction of late preeclampsia. *J. Matern. Fetal Neonatal Med*. **2019**, *32*, 3729–3733. https://doi.org/10.1080/14767058.2018.1471592.
26. Yang, L.; Sun, G.; Wang, A.; Jiang, H.; Zhang, S.; Yang, Y.; Li, X.; Hao, D.; Xu, M.; Shao, J. Predictive models of hypertensive disorders in pregnancy based on support vector machine algorithm. *Technol. Health Care* **2020**, *28* (Suppl. S1), 181–186. https://doi.org/10.3233/THC-209018.
27. Serra, B.; Mendoza, M.; Scazzocchio, E.; Meler, E.; Nolla, M.; Sabrià, E.; Rodríguez, I.; Carreras, E. A new model for screening for early-onset preeclampsia. *Am. J. Obstet. Gynecol*. **2020**, *222*, 608.e1–608.e18. https://doi.org/10.1016/j.ajog.2020.01.020.
28. Jhee, J.H.; Lee, S.; Park, Y.; Lee, S.E.; Kim, Y.A.; Kang, S.-W.; Kwon, J.-Y.; Park, J.T. Prediction model development of late-onset preeclampsia using machine learning-based methods. *PLoS ONE*. **2019**, *14*, e0221202. https://doi.org/10.1371/journal.pone.0221202.
29. Sovio, U.; Smith, G. Evaluation of a simple risk score to predict preterm pre-eclampsia using maternal characteristics: A prospective cohort study. *BJOG* **2019**, *126*, 963–970. https://doi.org/10.1111/1471-0528.15664.
30. Hou, Y.; Yun, L.; Zhang, L.; Lin, J.; Xu, R. A risk factor-based predictive model for new-onset hypertension during pregnancy in Chinese Han women. *BMC Cardiovasc. Disord*. **2020**, *20*, 1–10. https://doi.org/10.1186/s12872-020-01428-x.
31. Allen, R.; Aquilina, J. Prospective observational study to determine the accuracy of first-trimester serum biomarkers and uterine artery Dopplers in combination with maternal characteristics and arteriography for the prediction of women at risk of preeclampsia and other adverse pregnancy outcomes. *J. Matern. Fetal Neonatal Med*. **2018**, *31*, 2789–2806. https://doi.org/10.1080/14767058.2017.1355903.
32. Schaller, S.; Knippel, A.J.; Verde, P.E.; Kozlowski, P. Concordance-analysis and evaluation of different diagnostic algorithms used in first trimester screening for late-onset preeclampsia. *Hypertens. Pregnancy* **2020**, *39*, 172–185. https://doi.org/10.1080/10641955.2020.1750627.
33. Murtoniemi, K.; Villa, P.M.; Matomäki, J.; Keikkala, E.; Vuorela, P.; Hämäläinen, E.; Kajantie, E.; Pesonen, A.-K.; Räikkönen, K.; Taipale, P.; et al. Prediction of pre-eclampsia and its subtypes in high-risk cohort: Hyperglycosylated human chorionic gonadotropin in multivariate models. *BMC Pregnancy Childbirth*. **2018**, *18*, 1–10. https://doi.org/10.1186/s12884-018-1908-9.
34. Al-Rubaie, Z.T.A.; Hudson, H.M.; Jenkins, G.; Mahmoud, I.; Ray, J.G.; Askie, L.M.; Lord, S.J. Prediction of pre-eclampsia in nulliparous women using routinely collected maternal characteristics: A model development and validation study. *BMC Pregnancy Childbirth*. **2020**, *20*, 1–14. https://doi.org/10.1186/s12884-019-2712-x.
35. Antwi, E.; Klipstein-Grobusch, K.; Browne, J.L.; Schielen, P.C.; Koram, K.A.; Agyepong, I.A.; Grobbee, D.E. Improved prediction of gestational hypertension by inclusion of placental growth factor and

pregnancy associated plasma protein-a in a sample of Ghanaian women. *Reprod. Health* **2018**, *15*, 1–10. https://doi.org/10.1186/s12978-018-0492-9.
36. Pihl, K.; Sørensen, S.; Jørgensen, F.S. Prediction of preeclampsia in nulliparous women according to first trimester maternal factors and serum markers. *Fetal Diagn. Ther*. **2020**, *47*, 277–283. https://doi.org/10.1159/000503229.
37. Sandström, A.; Snowden, J.M.; Höijer, J.; Bottai, M.; Wikström, A.-K. Clinical risk assessment in early pregnancy for preeclampsia in nulliparous women: A population based cohort study. *PLoS ONE* **2019**, *14*, e0225716. https://doi.org/10.1371/journal.pone.0225716.
38. Allotey, J.; Laivuori, H.; Snell, K.I.; Smuk, M.; Hooper, R.; Chan, C.L.; Ahmed, A.; Chappell, L.C.; von Dadelszen, P.; Dodds, J.; et al. Validation and development of models using clinical, biochemical and ultrasound markers for predicting pre-eclampsia: An individual participant data meta-analysis. *Health Technol. Assess.* **2020**, *24*, 1. https://doi.org/10.3310/hta24720.
39. O'gorman, N.; Wright, D.; Syngelaki, A.; Akolekar, R.; Wright, A.; Poon, L.C.; Nicolaides, K.H. Competing risks model in screening for preeclampsia by maternal factors and biomarkers at 11–13 weeks gestation. *Am. J. Obstet. Gynecol*. **2016**, *214*, 103.e1–103.e12. https://doi.org/10.1016/j.ajog.2015.08.034.
